# Life Cycle Assessment of Rare Earth Elements-Free Permanent Magnet Alternatives: Sintered Ferrite and Mn–Al–C

**DOI:** 10.1021/acssuschemeng.3c02984

**Published:** 2023-08-29

**Authors:** Alessia Amato, Alessandro Becci, Alberto Bollero, Maria del Mar Cerrillo-Gonzalez, Santiago Cuesta-Lopez, Semih Ener, Imants Dirba, Oliver Gutfleisch, Valentina Innocenzi, Myriam Montes, Konstantinos Sakkas, Irina Sokolova, Francesco Vegliò, Maria Villen-Guzman, Eva Vicente-Barragan, Iakovos Yakoumis, Francesca Beolchini

**Affiliations:** † Department of Life and Environmental Sciences, Università Politecnica delle Marche, Ancona 60131, Italy; ‡ Group of Permanent Magnets and Applications, IMDEA Nanoscience, Madrid 28049, Spain; § Department of Chemical Engineering, Faculty of Sciences, Universidad de Málaga, 29071 Malaga, Spain; ∥ Fundación ICAMCYL, International Center for Advanced Materials and Raw Materials of Castilla y León, 24009 León, Spain; ⊥ MNLT Innovations PC, Kifisias Ave. 125−127, 11524 Athens, Greece; # Department Functional Materials, Material Science Faculty, Technical University of Darmstadt, 64287 Darmstadt, Germany; 7 SmartWaste Engineering S.r.l., Piazzale Monteluco di Roio, 67100 L’Aquila, Italy

**Keywords:** Permanent magnets, ferrite magnet, Mn−Al−C magnets, Nd−Fe−B magnets, life cycle assessment, sustainability

## Abstract

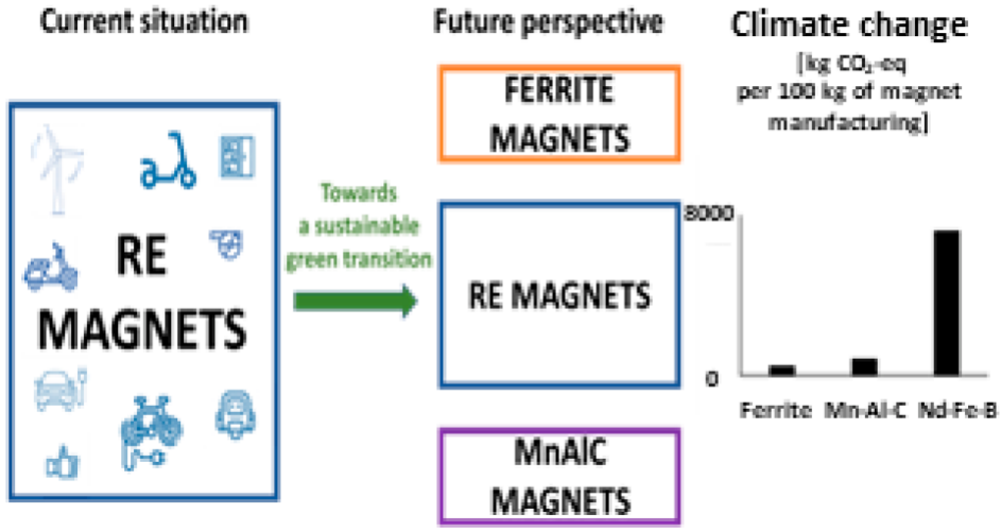

Permanent magnets are fundamental constituents in key sectors such as energy and transport, but also robotics, automatization, medicine, etc. High-performance magnets are based on rare earth elements (RE), included in the European list of critical raw materials list. The volatility of their market increased the research over the past decade to develop RE-free magnets to fill the large performance/cost gap existing between ferrites and RE-based magnets. The improvement of hard ferrites and Mn–Al–C permanent magnets plays into this important technological role in the near future. The possible substitution advantage was widely discussed in the literature considering both magnetic properties and economic aspects. To evaluate further sustainability aspects, the present paper gives a life cycle assessment quantifying the environmental gain resulting from the production of RE-free magnets based on traditional hexaferrite and Mn–Al–C. The analysis quantified an advantage of both magnets that overcomes the 95% in all the considered impact categories (such as climate change, ozone depletion, human toxicity) compared to RE-based technologies. The benefit also includes the health and safety of working time aspects, proving possible reduction of worker risks by 3–12 times. The results represent the fundamentals for the development of green magnets that are able to significantly contribute to an effective sustainable transition.

## Abstract 2

The paper quantifies the environmental sustainability resulting from the substitution of Nd−Fe−B magnets with RE-free technology in possible applications.

## Introduction

Permanent magnets (PMs) play an important role in the transition toward a decarbonized and clean energy system due to their application in technologies such as electric vehicles and wind energy.^1^ However, their sustainability is questioned since the strongest permanent magnets nowadays are based on rare earth (RE) elements, which are classified by the European Union as critical raw materials due to their high economic importance and high supply risk.^2,3^ Among RE magnets, Nd–Fe–B magnets are suitable for many applications where a high magnetic performance is required, mainly for large uses (e.g., wind turbines and electricity generators) thanks to their strength.^4^ RE mining has a huge environmental and human health impact, and around 98% of these elements used for European economy (86% considering the worldwide market) comes from China, which owns the monopoly and defines the market rules.^5^ In this regard, the crisis of 2011 caused a relevant increase of RE prices proving the market instability and the necessity to seek RE-free alternatives to make them a true enabler of the green transition reducing the consumption of these elements.^6^ Therefore, the current challenge is designing RE-free magnets competitive with RE magnets in specific applications such as industrial motors and vehicle motors (where the key factor is the maintenance of efficiency and torque-speed characteristics).^7,8^ Nowadays, the family of hexagonal ferrites, already implemented in multiple applications, is the candidate for a partial rare-earth magnets substitution thanks to the improved performance in combination with the possibility of optimizing the design of products to take full advantage from the enhanced properties.^9^ More recently, also the ferromagnetic Mn–Al-based compounds are gaining attention for their potential to become new hard magnetic materials commercially available.^10−12^

Hard M-type hexaferrite magnets, also known as ceramic magnets, are characterized by low cost, very good corrosion resistance, and high temperature stability (up to 250 °C).^13^ They are based on MFe_12_O_19_ (referred to as ferrite for the rest of the work) where M can be barium or strontium, and they were first suggested as permanent magnets materials in 1952 by Went et al.^14^ Since then, ferrite magnets have been implemented in numerous applications, occupying a dominant position on the permanent magnets market, reaching the current production of about 1 × 10^6^ tons per year (vs 1.4 × 10^5^ tons per year of RE magnets).^6,15^ Their relatively good magnetic properties and low price (compared to the RE magnets) resulted in an increased interest in ferrite magnets as a RE-free alternative. A proof of that is the numerous research focused on optimizing the sintering route of ferrite magnets to improve their magnetic performance and make them more competitive.^16,17^

Nowadays, sintered ferrite magnets available on the market are synthesized following the powder metallurgy processing route (i.e., thermal sintering) using as raw materials iron oxide (Fe_2_O_3_) and barium carbonate (BaCO_3_) or strontium carbonate (SrCO_3_). Basically, the thermal process involves three main stages: presintering, compacting, and sintering.^18^ However, this technique requires prolonged times and elevated temperatures, which promote an excessive growth of the ferrite grains reducing the coercivity.^16^ Although the elevated temperatures are necessary to densify the pieces and obtain magnets with high remanence, their effect on the coercivity makes it necessary to optimize the process to ensure an equilibrium between a controlled grain growth and a good densification. Therefore, after years of investigation, it has been demonstrated that the addition of sintering aids, as SiO_2_ and CaO, can control the grain growth and promote the densification, resulting in fully dense bulk magnets with good magnetic properties.^19−22^

Despite the improvement introduced into the traditional sintering route, the search for new and modified ways of densification of ferrite magnets has gained attention in the past decade. The aim of these new methods is reducing the sintering time and temperature or overall energy consumption, using a more efficient and faster heating process, or combining the heating with a pressure process. The most innovative ferrite sintering techniques under research can be classified into four main groups: microwave sintering, flash sintering, spark plasma sintering, and hydrothermal sintering.^17^ Ferrite magnets sintered using these novel techniques already yield good densities and competitive magnetic properties, as can be observed in Table 1, although these techniques remain mainly relegated to laboratory activities.

**Table 1:** Magnetic Properties of Magnets Processed by Different Techniques^a^

Reference	Composition	Sintering technique	*M*_s_ [A m^2^ kg ^–1^]	*M*_r_ [A (m^2^ kg^–1^)] (μ_0_M_r_) [T]	μ_0_*H*_c_ [kA/m]	(BH)_max_ [kJ/m^3^]
Hexaferrite magnet						
(18)	SrFe_12_O_19_	Conventional (Thermal sintering)		(0.38)	270.6	33.5
(37)	SrFe_12_O_19_ SiO_2_	Conventional (Thermal sintering)	54		135.2	
(38)	SrFe_12_O_19_·0.2%PVA·0, 6%SiO_2_	Ceramic processing route with two-step sintering	58	46	163.1	
(39)	SrFe_12_O_19_	Microwave-assisted calcination route	54.8	29.52	421.8	
(40)	SrFe_12_O_19_	Microwave sintering	50.4		437.7	
(41)	M-SrFe_12_O_19_	Microwave sintering	64		95.5	
(42)	SrM ferrite fine particles (1.0%La_2_O_3_, 0.1%Co_3_O_4_)	Spark plasma sintering		(0.32)	326.3	18.2
(43)	SrFe_12_O_19_	Spark plasma sintering	73.6	65.8	167.1	21.9
(44)	SrFe_12_O_19_	Hydrothermal: Sol–gel precursor coating technique	64.5		389.9	
(45)	SrFe_12_O_19_	Hydrothermal	72.2	44.76	175.1	9.5
Mn–Al–C magnets						
(32)	Mn_53_Al_45_C_2_	Casting + annealing + hot extrusion		(0.61)	214.9	49.0
(46)	Mn_55_Al_45_C_1_	Mechanical milling + powder compaction + annealing	119	41	119.4	6.2
(47)	Mn_56_Al_44_	Mechanical milling + spark plasma sintering + rapid thermal annealing	28		193.4	
(48)	Mn_53.5_Al_44.5_C_2_	Arc-melting + annealing + high energy ball milling + hHot compaction	(0.50)	28	262.6	4.8
(47)	Mn_53.5_Al_44.5_C_2_	Arc melting + annealing + high energy ball milling + microwave sintering + compaction	94	39	87.5	4.0
(49)	(Mn_54_Al_46_)_97.56_C_2.44_	Gas atomization powdering + annealing + compaction	90	39	270.6	
(34)	(Mn_57_Al_43_)C_1.1_	Gas atomization powdering + annealing + hot compaction	77	42.3	287.3	11.0
(50)	(Mn_54_Al_46_)_97.56_C_2.44_	Melting + melt spinning + annealing + crushing + compaction	122		103.5	
(51)	Mn_54_Al_44_C_2_	Melting + melt spinning + mechanical milling + spark plasma sintering	(0.55)	(0.31)	143.3	

a *M*_s_, saturation magnetization; *M*_r_, remanence; *H*_c_, coercivity; (BH)_max_, maximum energy product.

Recent works have shown that nanocomposite magnets offer a possibility to engineer magnetic properties by using a mixture of hard and soft phases.^23,24^ Therefore, in addition to improved densification, enhancing the maximum energy product (BH)_max_ of ferrite magnets by increasing the saturation magnetization *M*_s_ via the exchange-spring-magnet principle has been tried and, if successful, would have great technological and economical importance. For example, bulk composite magnets by using Al-doped Sr-hexaferrite SrAl_2_Fe_10_O_19_^25^ as the hard phase and environmentally friendly iron nitride α″-Fe_16_N_2_ nanoparticles^26^ obtained by hydrogen reduction of Fe_2_O_3_^27^ as the soft (semihard) phase were studied demonstrating that indeed adding α″-Fe_16_N_2_ leads to increased *M*_s_ and a slight increase in *M*_r_.^28^

Another alternative in the search of new RE-free permanent magnet is the aforementioned Mn–Al-based alloy.^29^ Although neither Mn nor Al is ferromagnetic, the metastable τ-MnAl phase found in the 47%–60% atomic composition interval exhibit ferromagnetism. This finding was done by Kono et al.,^30^ who studied the thermodynamics characteristics, structural, morphology, and magnetic properties of Mn–Al materials. It was found that the magnetic properties of Mn–Al alloy depend on their crystalline structure, while the rhombohedral and cubic phases are reported as paramagnetic material. The ε-phase is antiferromagnetic. The τ-phase is ferromagnetic with a high coercivity at room temperature (*H*_c_) of 240 kA/m and a maximum energy density of (BH)_max_ 55.7 KJ/m^3^.^31^ The BH parameter is essential for the estimation of magnet performance, since the higher the (BH)_max_ is, the smaller is the magnet volume to generate a given flux density.^1,8,13^ The τ-MnAl alloy is metastable and formed from ε-MnAl by quenching and annealing at temperatures between 500 and 700 °C. One of the problems of this transformation is the decomposition of τ-MnAl to the equilibrium β-Mn and γ_2_-Al_8_Mn_5_ phases with prolonged annealing,^30^ that can be avoided by adding a small amount of carbon (<2% atomic) which expands the stability space of the τ-phase.^32^

The conventional sintering route of the Mn–Al–C permanent magnet is a process of casting-solution heat treatment after melting the pure elements to homogenize the solution until the τ-phase is obtained. The process concludes with the extrusion of the ingot to obtain anisotropic Mn–Al–C magnets.^29^ The main problem of this sintering method is the difficulty to control the grain size and the coexistence of different phases, with the formation of large particles that affect the coercivity.^33^ In the last years, with the aim of improving the Mn–Al–C sintering process, a new method has been proposed based on a powder method, which does not require the production of an ingot by casting and its subsequent solution treatment.^29,34^ In this powder method, a mother alloy is melted and powdered through an atomization process (which leads to the formation of ε-phase), followed by the hot compaction of the powder; this method has been simplified to a single step process that allows the transformation of the ε-phase into the τ-phase and, simultaneously, the compaction of the powder to end with a high-density magnet.^34^ On one hand, the techniques to make the powder material precursor include mechanical milling, gas atomization, melt spinning, or spark erosion. Novel methodologies developed in the laboratory such as “flash milling”^35^ have allowed reducing the processing time of the Mn–Al–C powder and developing coercivity by using milling times ranging from seconds to a few minutes by comparison with traditional milling methods typically going to several hours. On the other hand, the techniques to compact the powder to fabricate bulk MnAl-based magnets involve hot compaction, hot deformation, hot extrusion, spark plasma sintering, and microwave sintering. Numerous studies have been focused on optimizing the combination of two-stage sintering to produce bulk MnAl-based magnets (Table 1) with promising results, although the magnetic properties achieved so far are inferior compared to the properties obtained in the casting/hot extrusion method.

The state-of-the-art method proves the relevance of RE-free magnets and the necessity of the development of new technologies able to enhance their properties. As has emerged from the literature, the main reason that has pushed the research is the risk of RE supply.^4,6,7^ Nevertheless, environmental and social aspects related to magnet manufacturing (often neglected) also deserve attention. The present paper aims at filling this gap by the assessment of the environmental gain reached by the production of traditional ferrite magnets and reported Mn–Al–C magnets as an alternative to Nd–Fe–B for some specific applications. Considering the relevance of health and safety aspects related to mining, and the risks connected with this activity,^5,36^ the authors compared the worker accidents due to the production of ferrite and Mn, with those related to Nd, the main elements within the three kinds of magnets.

The present paper aims to quantify the burdens resulting from the manufacture of different PMs to evaluate the possible gain resulting from the material substitution in the applications where it is possible. This kind of information, currently lacking (or patchy) in the scientific literature, is vital to identify the most effective ways in which to invest to pursue sustainability goals.

## Material and Methods

### Methods and Software

**Figure 1:**
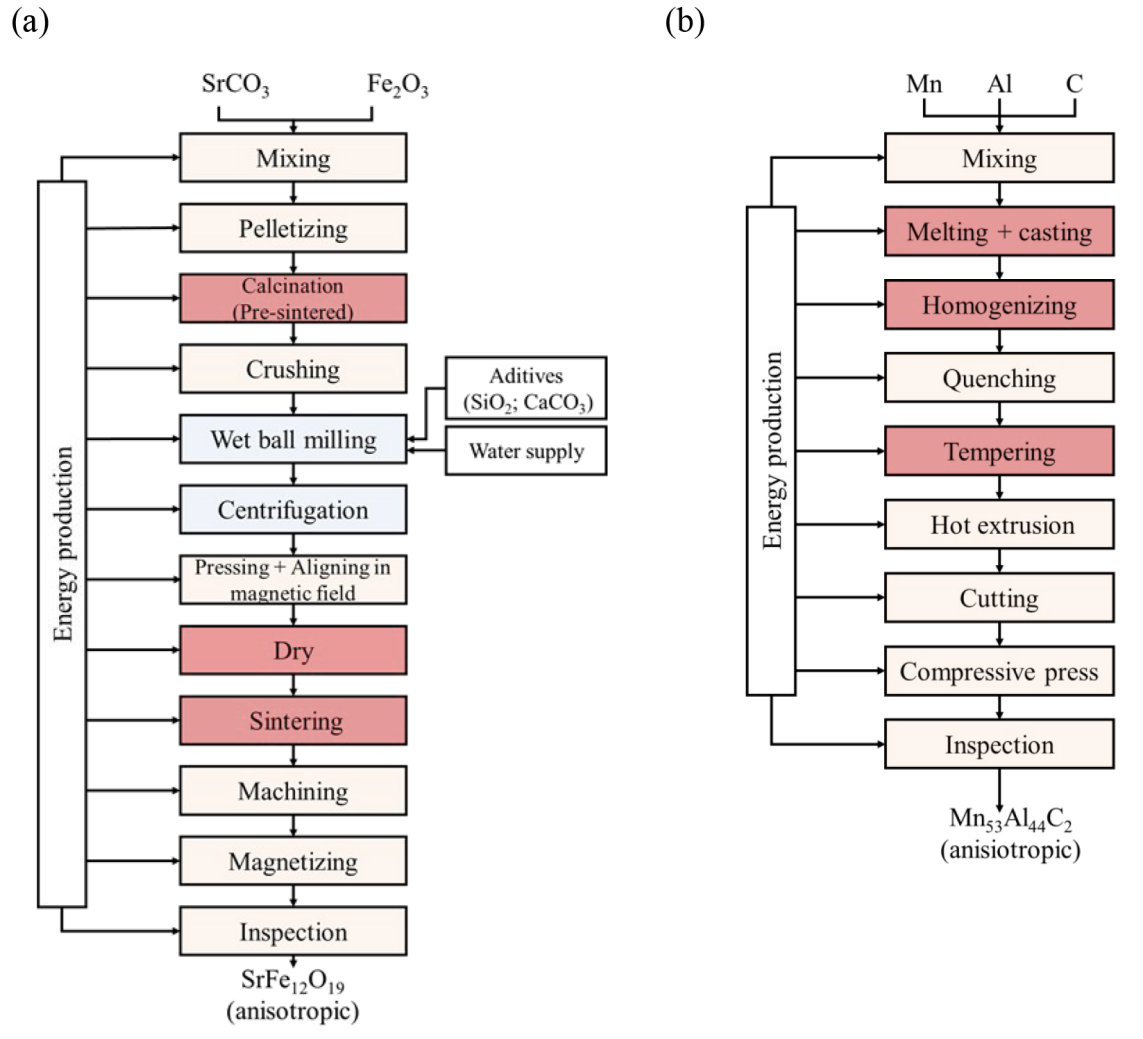
(a) Ferrite permanent magnet production route. (b) Mn–Al–C permanent magnet production route.

Considering the relevance of the RE-free magnets, the present paper aims at analyzing the sustainability of the manufacturing of both traditional ferrite magnets (industrially produced and commercially available) and Mn–Al–C magnets (not commercialized yet) as reported in the literature to assess strengths and weaknesses from an environmental point of view. With this aim, all the steps represented in Figure 1 (and the related mass and energy consumptions in Table S1, in Supporting Information) were considered. Mass balances were built following the details reported in patents selected from the worldwide platform Espacenet.^52^ The chosen patents were considered representative of magnets production since they comply with the process described by manufacturing companies.^53−55^Table S2 reports the details of machines selected to quantify the energy consumptions. Furthermore, considering the current trend toward the reduction of RE magnets use, the additional comparison with the sintered Nd–Fe–B magnet production was performed to assess the potential environmental gain resulting from the technology substitution (in applications where it is possible). The impact of the Nd–Fe–B magnet production process was estimated as the average value among those evaluated by Marx et al. and Arshi et al., in the common impact categories.^56,57^ They considered all of the production steps from the primary raw material mining to the magnet production. Considering these targets, a Life Cycle Assessment (LCA) methodology was applied in agreement with the LCA ISO standard 14040 and 14044:2006.^58,59^ The software used for data collection was Thinkstep Gabi 9.2.1.68, combined with the Database for Life Cycle Engineering. The method selected for the analysis (which included the classification, characterization, normalization and weighting steps) was Environmental Footprint 3.0, including all the environmental categories, recommended models at midpoint, together with their indicators, units, and sources.^60,61^ The same software was also used for health and safety of working time (HSWT) assessment focusing on the indicators of accidents for workers (both lethal and nonlethal), as one of the core goals of the life cycle working environment (LCWE) methods (implemented within the European Commission Project n° QLRT-1999-01298).

### Process Description

The present section describes the sintering routes used to obtain the RE-free magnets selected for the environmental assessment. The description includes energy and mass balances, referred to a functional unit of 100 kg of anisotropic magnet products (see Table S1).

#### Ferrite Thermal Sintering (Conventional)

Figure 1a shows the block diagram of the thermal sintering route to obtain ferrite magnet (anisotropic), based on the following patents: CN103265277B^62^ and CN108863335A.^63^ The process is comparable with those described by Nordelöf et al. and Sprecher et al., which were used to support the estimation of electricity consumption of the whole proposed process.^54,55^ The process starts with the mixture of raw materials (SrCO_3_, Fe_2_O_3_) followed by pelletization of the mixed powder. Considering the life cycle approach, the mixing step includes both the impact for energy consumption and that for SrCO_3_ and Fe_2_O_3_ (from mining to product production, including transports, emissions, waste, byproducts, and all the available information about supply chain). The related processes were extracted from Gabi database, as an average of the most relevant production processes worldwide (mainly placed in China mines and facility), implemented on the country norms basis. The pelletizing is performed to enhance the presintering step which takes place in a rotatory kiln at 1250 °C for 3 h to form metallic oxides. Once pellets have been calcined, the presintered pellets are ground into a coarse powder of 10 μm, and then, additives and water are added to the coarse powder and subjected to a secondary ball milling to obtain a fine slurry. Before the molding step, the slurry is dehydrated by centrifugation to the adjust the slurry concentration in 75 wt % and optimize the wet pressing. The fine slurry is molded under a pressure of 10 MPa and a DC magnetic field, to obtain cylinders with a diameter of 50 mm and length of 10 mm. The compacted magnets are placed in an oven at 75 °C for 24 h, for the impurity removal, before being sintered in a wagon kiln at 1230 °C for 8 h to fuse particles together and form dense solid material. Finally, the ferrite magnets produced are magnetized until saturation and inspected prior to their sale. The magnetic properties obtained in this sintering route are the following: *B*_r_ of 0.47 T, *H*_c_ of 239 kA/m, and (BH)_max_ of 35 kJ/m^3^.

#### Mn–Al–C Production Route

A Mn–Al–C permanent magnet is produced by melting and casting followed by a hot extrusion method described in EP0034058A1^31^ (Figure 1b). The mixing block includes both energy and raw material (Mn, Al, C) mining and productions, in agreement with that described for the ferrite magnet. After melting the pure elements in an induction furnace at 1400 °C, the molten mixture consisting of 70 wt % Mn, 29.5 wt % Al, and 0.5 wt % C is molded into the shape of cylinders with 40 mm diameter and 30 mm height. The ingots are homogenized at 1100 °C for 2 h to obtain the Mn–Al–C ε-phase, followed by air quenching and tempering at 600 °C for 20 min to promote the ε→τ phase transformation. The tempered pieces are extruded at 720 °C with a pressure of 780 MPa to a diameter of 15 mm, causing a final ingot with a length of 220 mm. Thereafter, each ingot is cut into pieces with a thickness of 20 mm to be subjected to compressive working, reducing 20% the height of the cylinders. The final sintered Mn_53_Al_45_C_2_ permanent magnets, with dimensions of 15 mm in diameter and 16 mm in height, have the following magnetic characteristics: *B*_r_ of 0.47 T, *H*_c_ of 239 kA/m, and (BH)_max_ of 35 kJ/m^3^.

## Results

### Sustainability Assessment of RE-Free Permanent Magnets

The results of classification and characterization steps of LCA allowed us to assess the impacts caused by the manufacture of sintered ferrite magnets (conventional production route) and Mn–Al–C magnets (not available in the market), considering three groups of categories: environmental conservation, resource depletion, and human health.

**Figure 2:**
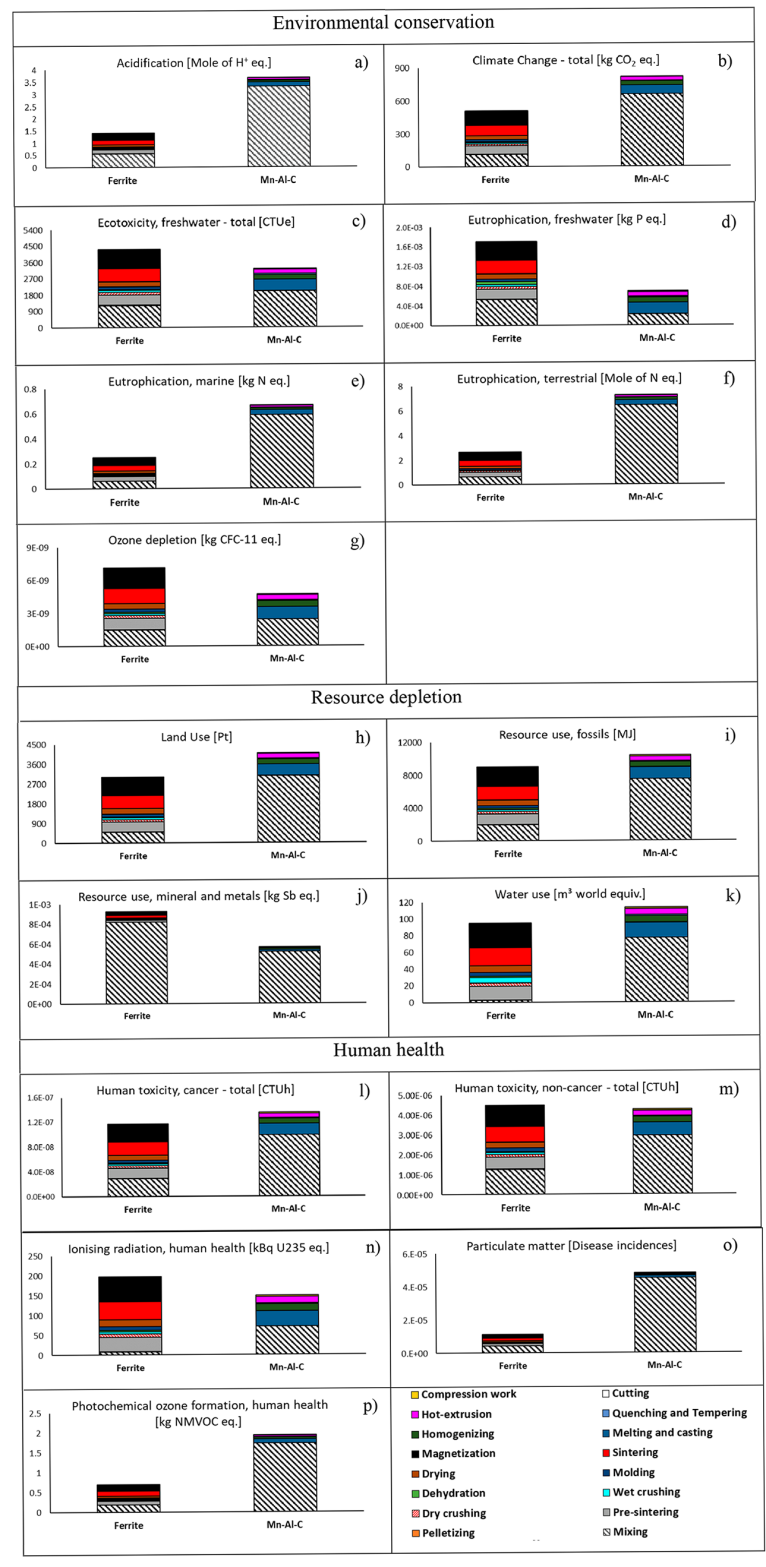
Assessment of environmental impacts in the categories of (a) acidification, (b) climate change, (c) ecotoxicity, freshwater, (d) eutrophication, freshwater, (e) eutrophication marine, (f) eutrophication terrestrial, (g) ozone depletion, (h) land use, (i) resource use, fossils, (j) resource use, mineral and metals, (k) water use, (l) human toxicity, cancer, (m) human toxicity, noncancer, (n) ionizing radiation, human health, (o) particulate matter, and (p) photochemical ozone formation, comparison between production processes of ferrite and Mn–Al–C magnets (functional unit: 100 kg of magnets).

Figure 2 highlights the relevance of the raw materials within the mixing step, irrespective of the impact category, mainly in the Mn–Al–C magnet. The reason is the raw material consumptions, considered as input of this process phase. The LCA allows us to consider all the steps of raw material production, including mining, extraction, refining, and transportation of the elements within the magnets. In this regard, ferrite shows an average contribution of 85% of the mixing impact, in all the considered categories, except for resource use, mineral, and metals, where 96% of the load is due to SrCO_3_ (Figure 2j). For the Mn–Al–C magnets, the mixing impact (which includes both the emissions for the necessary energy and those for the mining and refining of input raw materials) is almost completely due to Mn (with an average contribution in mixing around 65%) and Al (34%). The Al supply represents the main issue for ozone depletion and ionizing radiation and human health (Figure 2g, n) due to the conditions of the production process from bauxite by the Bayer chemical process and refining by electrolysis. (The Al process production, described in the software database, is representative of the most common European primary production, in agreement with the European Aluminum profile report of 2018. It includes agent recirculation and scrap recycling.)^2^ The results related to raw materials highlight the relevance of recycling strategies in the magnet field, already discussed in the literature,^64−66^ proving that possible substitution of elements from secondary resources could potentially decrease the process impacts more than 60%, on the impact category basis (compared to the RE primary production^66^).

Considering the operative conditions of magnet manufacturing, the environmental burden related to the electricity consumption is relevant, and it represents over half the impact of ferrite magnets. For this evaluation, an average European grid mix has been considered, where the main energy resources are nuclear (25%), natural gas (20%), hydro (12%), and wind (12%). In more detail, the energy consumption explains the impact of the steps of pre/sintering, and magnetization of ferrite magnet, almost equally distributed among the three phases, in all categories. On the other hand, the steps of pelletizing, crushing, dehydration, molding, and drying show environmental burdens lower than 10% in all the considered categories. The electricity consumption of melting and casting of the Mn–Al–C magnet (215 kWh per 100 kg of magnet) explains the impact of this step on the whole process load, with an average contribution of 12%. This percentage reaches values around 30% in the categories of eutrophication freshwater, ozone depletion, and ionizing radiation (Figure 2 d, g, n). In the same categories, particularly sensitive to energy consumption, the homogenizing (94 kWh) and hot-extrusion (84 kWh) contributions are highlighted, around 10%. Both cutting and compression work do not affect the whole analysis.

**Figure 3:**
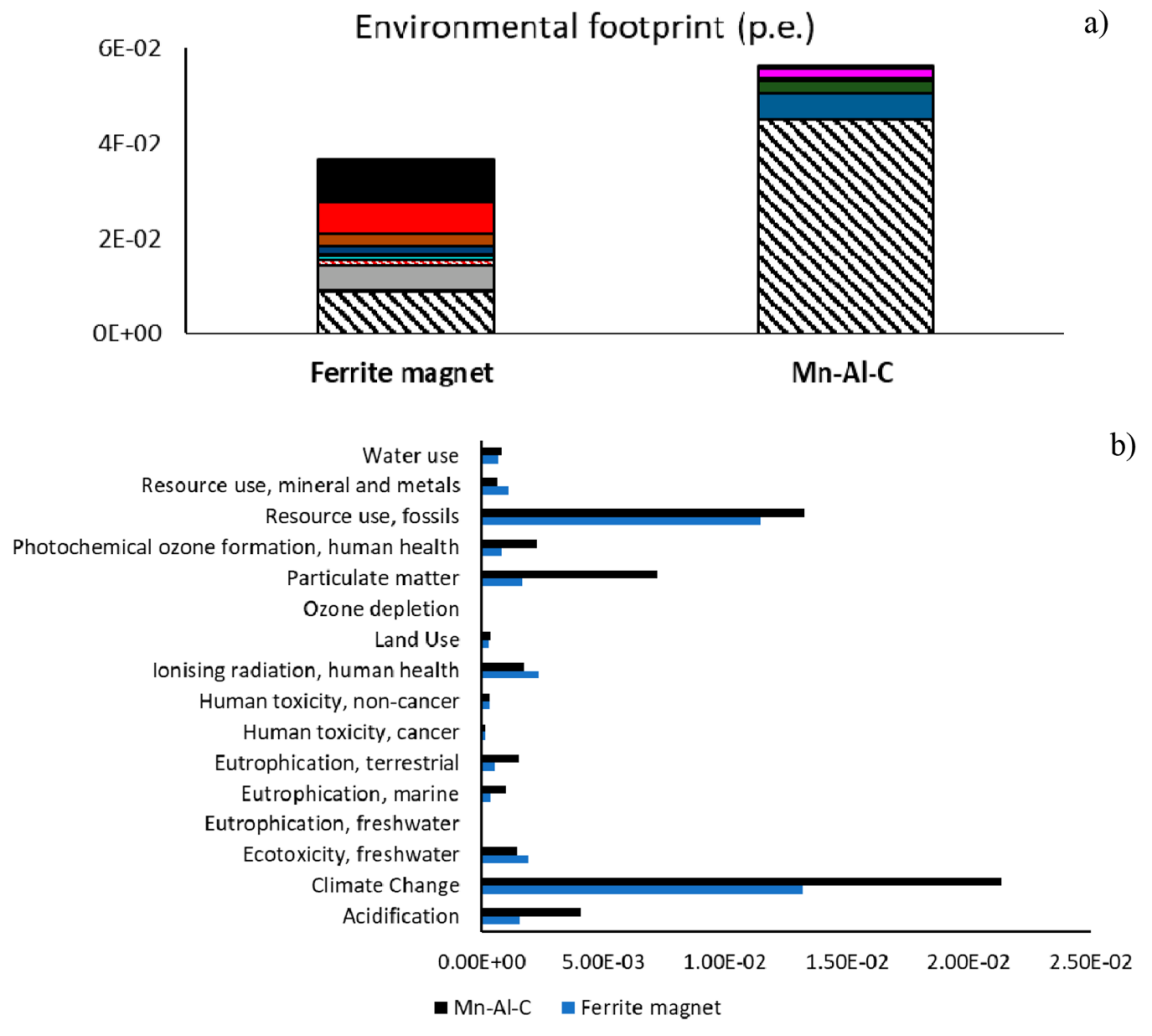
Assessment of environmental footprint: (a) comparison between production processes of ferrite and Mn–Al–C magnets and (b) contribution of impact categories (functional unit: 100 kg of magnets). The legend of (a) is the same of Figure 2.

The heterogeneity of the results in the different impact categories does not allow considerations about comparison between the two processes. Furthermore, each category shown in Figure 2 is expressed by a specific category indicator and a whole conclusion is not possible. For these reasons, the steps of normalization (calculating the magnitude of category indicator results relative to reference information) and weighting (converting and possibly aggregating indicator results across impact categories using numerical factors based on value-choices) play an essential role.^59^Figure 3a shows an impact savings around 35% of sintered ferrite magnets (assessed as person equivalent, i.e., the number of European people (average citizens) that generates the same environmental effect in one year, in terms of impacts from global to local as well the resource consumptions^67,68^). The reason for this whole result is better explained in Figure 3b which identifies the most critical impact categories, mainly climate change, particulate matter, and resource use (fossil fuel), where the lowest impact of ferrite was estimated (Figure 2b, i, o). The environmental footprint resulting from normalization and weighting confirms the raw material supply issues, mainly for Mn–Al–C magnets. The highest impact of ferrite manufacturing in the category of ionizing radiation is justified by the higher electricity consumption of ferrite production than that of the Mn–Al–C one (visible in Figure 2n and 3b). In more detail, the effect of ionizing radiation on human health is caused by the radionuclides (potentially toxic for humans) produced by both the nuclear energy production, and the mineral oil and gas extraction, used as energy carrier.^69,70^ Considering the energy issue, a winning solution is process improvement by feeding it with renewable resources. Some estimations performed by Gabi software proved that electricity production, by photovoltaic or wind, is able to decrease the energy footprint by about 90%.

### Environmental Sustainability and Health and Safety Assessment RE-Free vs Nd–Fe–B Magnets

**Figure 4:**
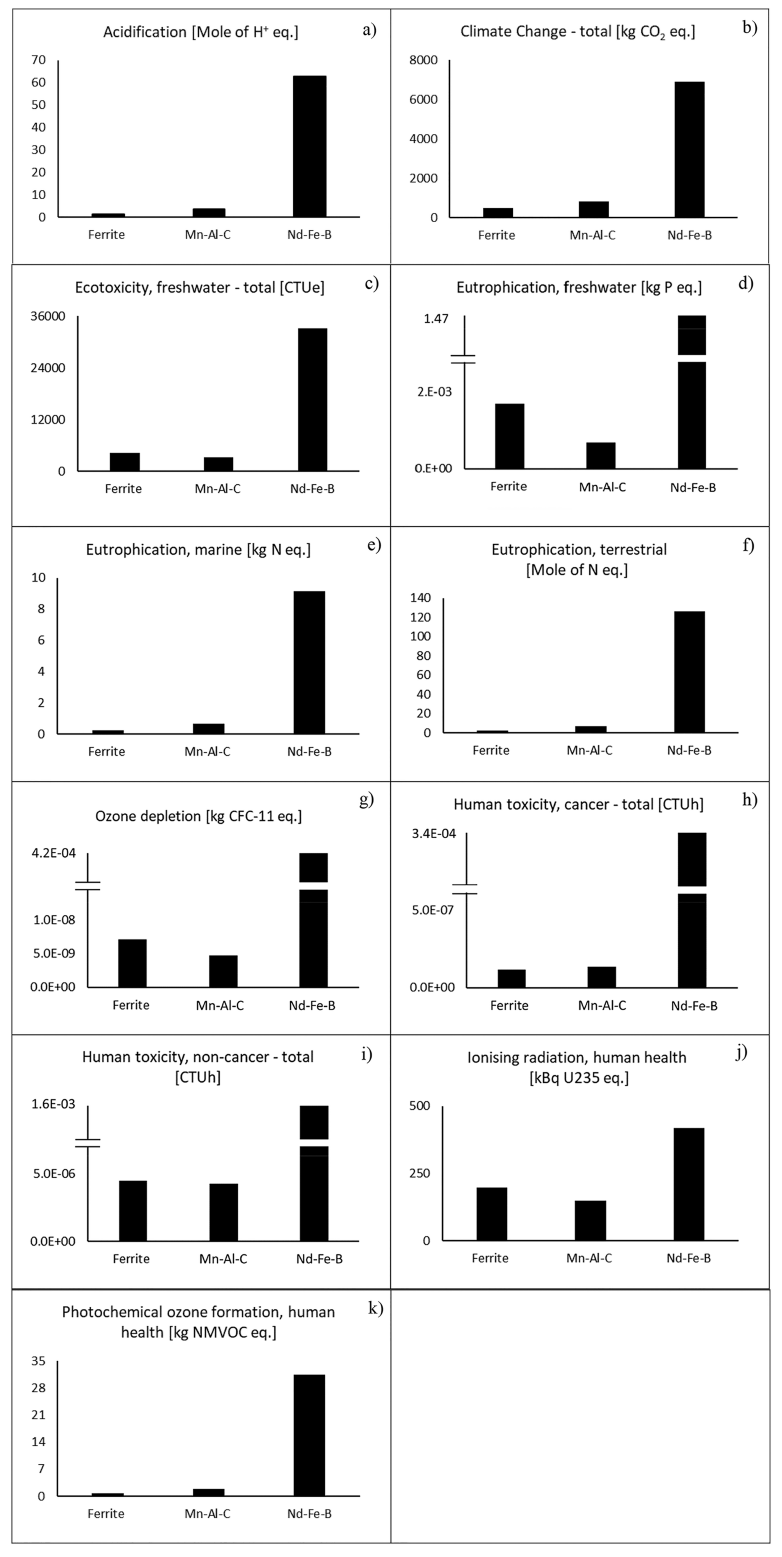
Environmental impact assessment of manufacturing processes in the categories of (a) acidification, (b) climate change, (c) ecotoxicity, freshwater, (d) eutrophication, freshwater, (e) eutrophication marine, (f) eutrophication terrestrial, (g) ozone depletion, (h) human toxicity, cancer, (i) human toxicity, noncancer, (j) ionizing radiation, human health, and (k) photochemical ozone formation, comparison among sintered ferrite, Mn–Al–C, and Nd–Fe–B magnets.

LCA represents an excellent tool to assess the possible environmental gain resulting from the substitution of RE magnets with alternatives such as ferrite and Mn–Al–C (in technologies where it is possible). In this regard, Figure 4 shows the comparison between the environmental impacts estimated for RE-free magnets and those resulting from Nd–Fe–B manufacturing (extracted from the literature, as average value of several process options, (BH)_max_ 358 kJ/m^3^).^56,71^ It is evident that the environmental burden of Nd–Fe–B far exceeds (also many orders of magnitude in the categories of eutrophication freshwater, ozone depletion, and human health Figure 4d, g–i) the other technologies in all the impact categories considered. The results show possible emission savings of 93% and 87% by using sintered ferrite and Mn–Al–C, respectively, in the category of climate change, considered one of the most important environmental issues, at the moment (Figure 4b). This advantage is explained by the avoided use of Nd and Dy which cause about 50% of the whole process emission of CO_2_-eq due to both chemicals and energy used in several processing steps (such as solvent extraction, electrolysis, and leaching). For the same reasons, their contribution to the process impact reaches up to 98% in the category of eutrophication marine, as explained by Marx et al.^56^ The advantage of both ferrite and Mn–Al–C magnets is strongly supported by the possibility to compare their impacts with those of Nd–Fe–B estimated by different methods, software, and mining sites. Indeed, Figure 4 reports the average value of the environmental burden quantified by different authors,^56,57^ but a benefitsof RE-free magnets, higher than 90%, is confirmed by the comparison with the scenario with the lowest impacts (described by Arshi et al. which considers the Bayan Obo RE deposit. The main difference is observed in the category of marine eutrophication, where the environmental advantage of RE-free magnets decreases up to 50% and 40% for ferrite and Mn–Al–C ones, respectively.

Considering the three spheres of sustainability, environmental (discussed above), economic (widely discussed in the literature), and social, the addition of some considerations about social aspects is relevant. The negative effect of RE mining for the population has been documented by both scientific literature and Greenpeace China, which told about illegal activities, terrible effects on population health due to pollutant release in soil, water, and air, production of hazardous radioactive waste, and poor working conditions due to the lack of regulations.^72−77^ Starting from this observation, the evaluation of the HSWT focusing on the indicators of lethal and serious and nonlethal accidents was performed considering the production of ferrite, manganese, and neodymium used for the manufacture of the three kinds of magnets included in the present study. The results showed that the cases of accidents (both lethal and nonlethal) due to Nd supply are 12 and 3 times higher than ferrite and Mn, despite the highest consumption (89 kg of ferrite, 72 kg of Mn vs 27 kg of Nd, reported in the mass balances in Supporting Information, Table S1) used for the production of 100 kg of magnets.

### Sensitivity Analysis: Effect of Electricity Grid Mix

The described results can be affected by several variables such as the geographical area where the magnet manufacture is implemented. Considering the relevance of energy consumption, the variability due to the process location and the consequent characteristics of the electricity grid mix were evaluated by a sensitivity analysis. With this aim, the results described in Figures 2 and 3, where an average European grid mix was selected, were compared to the same scenarios supplied by a Chinese grid mix. This country is selected since it is one of the main leaders in raw material supplying and processing, also in the permanent magnet field.^2,78^ The Chinese grid mix is completely different from the European one, characterized by the main contributions of hard coal (63% of the whole energy supply) and hydroelectric (17%). As reported in Figure S1, the change in electricity supply mix causes relevant effects on many environmental categories. The effect on climate change is particularly remarkable for the high weight of this category in normalization and weighting phases (as proven in Figure 3). As shown in Figure S1b, the hard coal use produces a negative effect on the ferrite manufacturing process, mainly on the energy-intensive steps of magnetization and sintering, making the whole impacts of ferrite and Mn–Al–C magnets comparable. This aspect justifies the results in Figure S2, related to the normalized and weighted burden, which shows an increase of 40% for the ferrite scenario and of 15% for the Mn–Al–C one. Despite the electricity grid mix variation, the advantage of both magnets, compared to Nd–Fe–B technology, is evident in Figure S3, confirming the environmental gain resulting from the replacement of RE magnets, by free-RE magnets, in applications where it could be possible (irrespective of the geographical placement Europe vs China).

## Discussion

The results shown in the Results section prove the possible advantage resulting from the substitution of RE magnets by RE-free magnets, not only to respond to the raw material supply risk but also to contribute to global sustainability. These achievements are further confirmed by the sensitivity analysis which showed the greatest sustainability of both ferrite and Mn–Al–C magnets, compared to RE magnets, irrespective of the country where the manufacture takes place. The results are relevant especially in the perspective of the increase of Nd–Fe–B magnet demand (estimated up to 70% by 2040^13^). It is evident that this substitution cannot be hypothesized for all the fields and applications, considering the best performance of the RE magnet. Nevertheless, ferrite and Mn–Al–C magnets could represent the best option for less energy-intensive applications such as power window/seats in vehicles, switches, fans, blowers in appliances, some power tools, and loudspeakers and buzzers in electro-acoustic devices,^13^ but also in the motors of some e-vehicles (e-scooter, e-bike, and e-motorbike) by taking advantage of the possibility of an easy implementation of redesigns.^9^ Another aspect to take into account is that ferrite magnets show much lower electrical conductivity than Nd–Fe–B magnets; that is translated into lower losses due to eddy currents, thus reducing demagnetization phenomena.^79^

These aspects, combined with the obtained sustainability results, prove that it is worth investing in scientific and technological research to enhance the properties and applications of RE-free magnets properties and applications. In this regard, Zhao et al. developed two innovative traction motors for hybrid vehicles with ferrite magnets with competitive torque density and efficiency as well as operating range compared to a RE magnet motor used in the commercialized third-generation Toyota Prius.^80^ Promising results are also achieved by the European Project Motorbrain, in which 30 partners from 9 European countries worked with the aim of increasing the range and reliability of electric vehicles while reducing the dependence of Asian REs. With this aim, the optimized ferrite magnets with a similar or better power density than an equivalent induction motor were developed.^7,81^ Furthermore, De Gennaro et al. explained that the reduced performance of motors of hybrid vehicles, due to the avoided RE use, can be compensated by an enhanced electromagnetic and mechanical design of the stator and rotor components.^5^ The research and technological activities developed in the framework of the European Project NANOPYME (2012–2015)^82^ reached successful design, construction, and testing of a novel fully ferrite-based motor;^83,84^ this project went one step further and succeeded in the integration of the motor in a fully operational e-motorbike demonstrating its functionality under real use conditions. The most recent project, AMPHIBIAN (2017–2019), improved the ferrite properties by metallic nanowire to produce a patented flywheel, a mechanical device able to store energy.^85^ Furthermore, the ReFreeDrive Horizon 2020 project (2017–2021) worked on the development of RE-free next-generation electric drivetrains for fully electric vehicles.^86^ These important achievements reinforce the hypothesis considered in the present paper to substitute a RE magnet with a ferrite one, with the aim to fit the sustainability target. As concerns Mn–Al–C magnets, promising results were obtained in the field of RE-free magnets.^87,88^ In this regard, Palmero et al. described an innovative technology in which gas-atomized MnAlC particles, combined with polymer, were used to produce scalable RE-free permanent magnet composites and extruded flexible filaments, useful for 3D-printing.^89^ Noteworthy is that the ongoing European project PASSENGER, which is working on both improved Sr-ferrite and MnAlC magnets as substitutes of RE-magnets, focuses on several applications. The project identified five core products where the RE-free magnets could penetrate the market, particularly e-bikes, e-motorbikes, e-cars, e-scooters, and pump drives). The positive effect that these conversions could have on the market is highlighted by the growing market of these products. The forecasts talk about growth of 60% of the e-bike market,^90,91^ 50% of the e-motorbike market,^92^ 70% of the e-car market,^93,94^ and 25% of the e-scooter market^95^ between 2021 and 2031. Concerning pump drives, the estimations report around a 7.6% annual growth rate in the same decade.^96,97^ Only considering the e-bike market, the Confederation of the European Bicycle Industry reported around 5 million e-bikes in 2021,^98^ each unit with 300 g of permanent magnets;^99^ therefore, the expected market growth between 2021 and 2031 can be translated into 62 ktons of CO_2_-eq (considering the average values used for the estimations in Figure 4).

## Conclusions

The current world crisis due to raw material and energy supplies has pushed research toward the development of alternative strategies able to ensure the economic stability of Europe. In addition to the market benefit, the present paper proves that the substitution of RE-rich permanent magnets with RE-free magnets (in technologies where it is possible) could represent an environmental and social gain. The LCA analysis quantified an advantage of both conventional sintered ferrite and prototype Mn–Al–C magnets that demonstrate 95% improvement, compared to Nd–Fe–B magnets, in many environmental categories irrespective of the geographical area where it takes place. Indeed, considering the most relevant category of climate change, the estimation showed that the manufacturing of 1 kg of ferrite and Mn–Al–C magnets causes impacts around 5.1 and 8.2 kg CO_2_-eq, respectively, if performed in Europe, and 9.7 and 10.1, if performed in China, compared to the 69 kg CO_2_-eq for each kg of Chinese Nd–Fe–B magnet (chosen as baseline scenario). The positive effects were also estimated considering health and safety aspects, with a possible reduction of worker risks of between 3 and 12 times.

Considering the increasing necessity of green technologies and the consequent growing demand of raw materials, the obtained results represent excellent food for thought to understand that an effective sustainable conversion needs a broad view. Only conversions from gasoline cars to hybrid/electric vehicles or transitions from nonrenewable to renewable energy are not enough. The whole life of these technologies, including the choice of raw materials for their production, must be assessed in the perspective sustainability. In this regard, the possibility to enhance technology RE-free, for all the possible applications, turned out to be a winning strategy for earth conservation and the reduction of population risks.

The current study looks at the three different magnet types in isolation from their respective target device applications. Future studies should focus on specific technologies combining sustainability aspects with particular technical issues. For example, to achieve comparable power output and torque, a ferrite magnet motor needs to be larger and heavier, using more magnet material, more steel, and more copper for windings. This will inevitably modify the LCA balance, and the consideration of a single component (e.g., magnet) could be limiting. In these cases, the system boundary growth, adding the entire product (e.g., electric motor or generator), could provide a more realistic picture of the resultant environmental, ecological, economic, and social implications.

In this context, the constant interaction between industrial and research realities represents the key factor for the development of successful strategies that involve the permanent magnet life cycle. The present paper is proof of the relevance of the multiple expertise joining among the partners of European Horizon project PASSENGER to evaluate different aspects of magnet manufacturing, from the technical relevance of the processes to the sustainability aspects, with the aim to evaluate the most promising options for the future.

## Supporting Information Available

Mass and energy balances used for LCA, sensitivity analysis, and effect of electricity grid mix (PDF)
