# Supplementary material for: Life Cycle Assessment of Rare Earth Elements-Free Permanent Magnet Alternatives: Sintered Ferrite and Mn–Al–C
Source: ACS Sustain Chem Eng. 2023 Aug 29;11(36):13374–86. doi: 10.1021/acssuschemeng.3c02984 (PMC10498492; doi:10.1021/acssuschemeng.3c02984)
Supplement: Supplementary file 1 — sc3c02984_si_001.pdf [file sc3c02984_si_001.pdf]

Supporting information

Life cycle assessment of rare earth elements-free permanent magnet alternatives: sintered ferrite and Mn-Al-C

Alessia Amato [1*], Alessandro Becci [1*], Alberto Bollero [2], Maria del Mar Cerrillo-Gonzalez [3], Santiago Cuesta-Lopez [4], Semih Ener [6], Imants Dirba [6], Oliver Gutfleisch [6], Valentina Innocenzi [7], Myriam Montes [4] Konstantinos Sakkas [5], Irina Sokolova [4], Francesco Vegliò [7], Maria Villen-Guzman [3], Eva Vicente-Barragan [4], Iakovos Yakoumis [5], Francesca Beolchini [1]

[1]Department of Life and Environmental Sciences, Università Politecnica delle Marche, Ancona, 60131, Italy

[2]Group of Permanent Magnets and Applications, IMDEA Nanoscience, Madrid, 28049, Spain

[3]Department of Chemical Engineering, Faculty of Sciences, Universidad de Málaga, 29071, Malaga, Spain

[4]Fundación ICAMCYL, International Center for Advanced Materials and Raw Materials of Castilla y León, León, Spain

[5]MNLT Innovations PC, Kifisias Ave. 125-127, 11524, Athens, Greece

[6]Department Functional Materials, Material Science Faculty, Technical University of Darmstadt, 64287 Darmstadt, Germany

[7]SmartWaste Engineering S.r.l. Piazzale Monteluco di Roio, 67100 L'Aquila, Italy

*Corresponding authors:

Alessia Amato, a.amato@univpm.it, +39 0712204225

Alessandro Becci, a.becci@univpm.it, +39 0712204225

Number of pages: 10
Number of tables: 2
Number of figures: 3

Table S1: Energy and mass balance of the two sintering routes (based on the production of 100 kg of final permanent magnet. *The quantity of dispersants, lower than 1% of the whole input, make them negligible for the analysis purpose).

Process	Step	Input	Output
Ferrite thermal sintering	Mixing	0.5 kWh<br>13.3 kg $SrCO_3$<br>89.0 kg Fe_2O_3<br>0.09 kg SiO_2<br>0.2 kg H_3BO_3	102.6 kg mixed powder
	Pelletizing	8.4 kWh	107 kg ball pellet (diameter 6 mm)
	Pre-sintering	195 kWh	96.2 kg $SrFe_{12}O_{19}$-SiO_2<br>2.4 kg Fe_2O_3<br>4.0 kg CO_2
	Dry crushing	44 kWh	Pre-sintered powder (<10 µm)
	Wet crushing	33 kWh<br>95.9 kg water<br>1.0 kg $CaCO_3$<br>0.1 kg SiO_2<br>0.3 kg dispersants*<br>0.1 kg H_3BO_3	195.9 kg slurry
	Dehydration	18.5 kWh	133.3 kg slurry (75 wt.% solid)<br>62.6 kg water
	Moulding	55 kWh	1400 unit of compact magnet<br>(Diameter 5 cm; heigh 1 cm; ρ: 3.8 g cm^{-3})
	Drying	96 kWh	1400 unit (=100 kg solid)<br>33.3 kg water
	Sintering	250 kWh	1400 units synthetized ferrite magnet
	Magnetization	350 kWh	1400 units ferrite PM (=100 kg)<br>B_r 0.43 T; H_c 310 kA/m; $(BH)_{max}$ 35 kJ/m^3
Mn-Al-C sintering route	Mixing	0.5 kWh<br>72 kg Mn<br>29.5 kg Al<br>0.5 kg C	102 kg mixed powder<br>(72 kg Mn, 29.50 kg Al; 0.5 kg C)
	Melting and casting	215 kWh	100 kg molten solution casting in cylinders<br>(70 wt% Mn; 29.5 wt% Al; 0.5 wt% C)<br>2 kg Mn
	Homogenizing	94 kWh	100 kg ε-phase Mn-Al-C ($Mn_{53}Al_{45}C_2$)
	Quenching and Tempering	18 kWh	100 kg τ-phase Mn-Al-C<br>(D: 4 cm; H: 3 cm; ρ: 5.1 g cm^{-3})<br>(Equivalent to 520 billets)
	Hot-extrusion	84 kWh	100 kg τ-phase Mn-Al-C<br>(D: 1.5 cm; H: 22 cm)

	Cutting	-	100 kg τ-phase Mn-Al-C (D: 1.5cm; 2 cm) Equivalent to 5720 units
	Compression work	19 kWh	100 kg τ-phase Mn-Al-C (D: 1.5; H: 1.6 cm) 5720 units Mn-Al-C magnet B_r 0.47 T; H_c 238.7 kA/m; $(BH)_{max}$ 35 kJ/m^3

Table *S2*: Characteristics of machine selected for the processes.

**Process**	**Step**	**Capacity**	**Reference**
Ferrite thermal sintering	Mixing	100L/batch	[1]
	Pelletizing	100 kg/h	[2]
	Pre-sintering	120 kg/h	[3]
	Dry crushing	200 kg/h	[4]
	Wet crushing	200 kg/h	[4]
	Dehydration	200 kg/h	[5]
	Moulding	100 kg/h	[6]
	Drying	270 kg/h	[7]
	Sintering	(2.5 kWh/kg)	[8]
	Magnetization	-	[8,9]
Mn-Al-C sintering route	Mixing	100L/batch	[1]
	Melting and casting	100 kg/h	[10]
	Homogenizing	400 L	[11]
	Quenching and Tempering	120 kg	[11]
	Hot-extrusion	-	[12]
	Cutting	-	
	Compression work	-	[13]

Environmental conservation

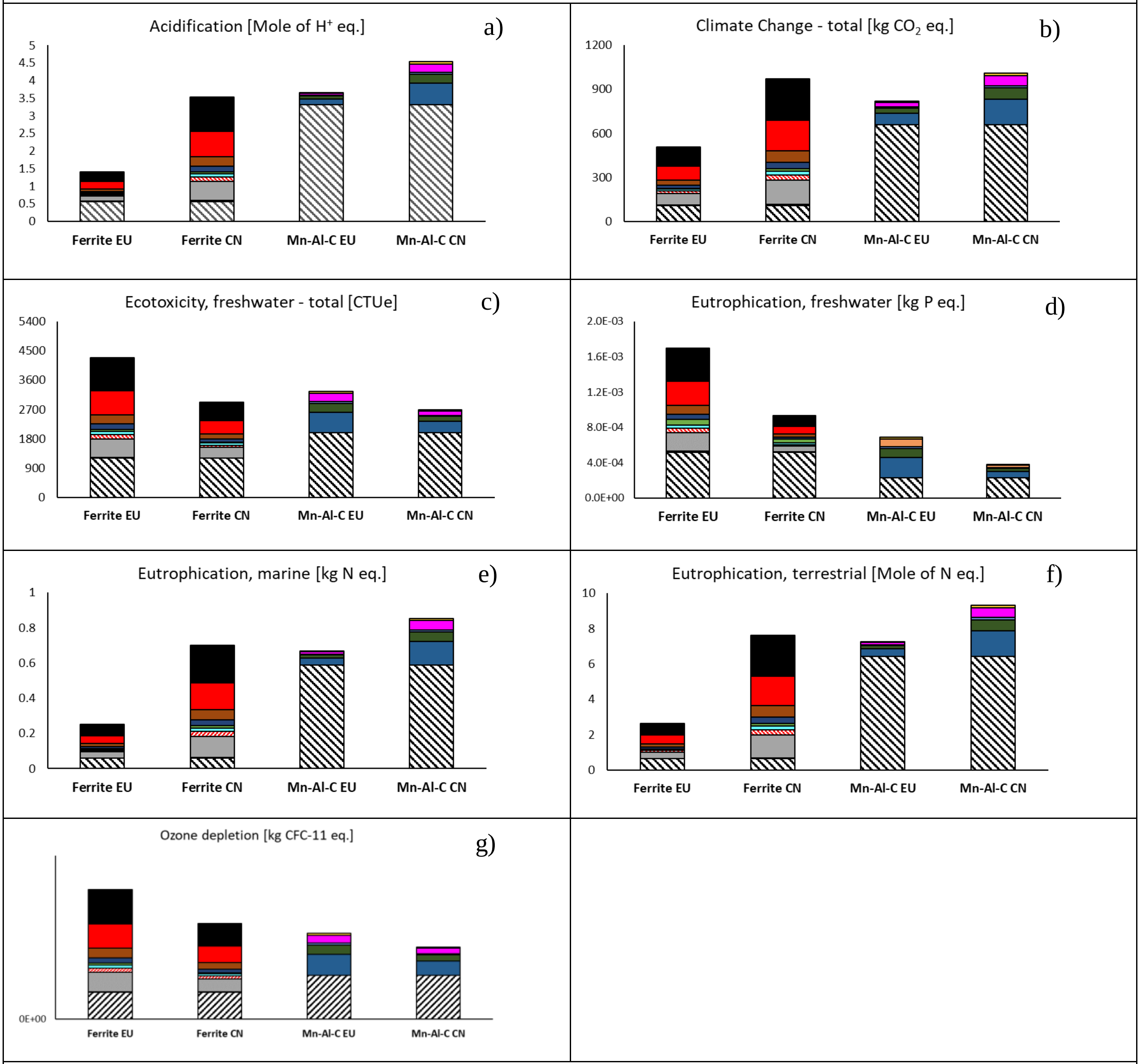

Resource depletion

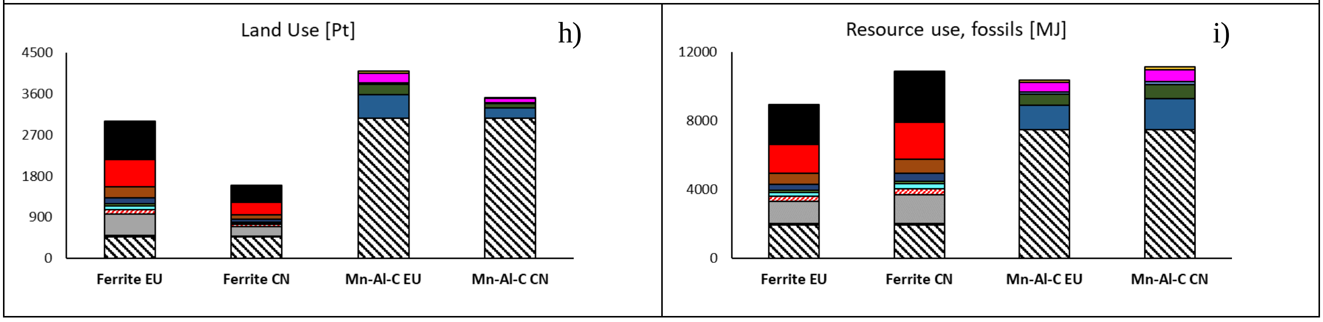

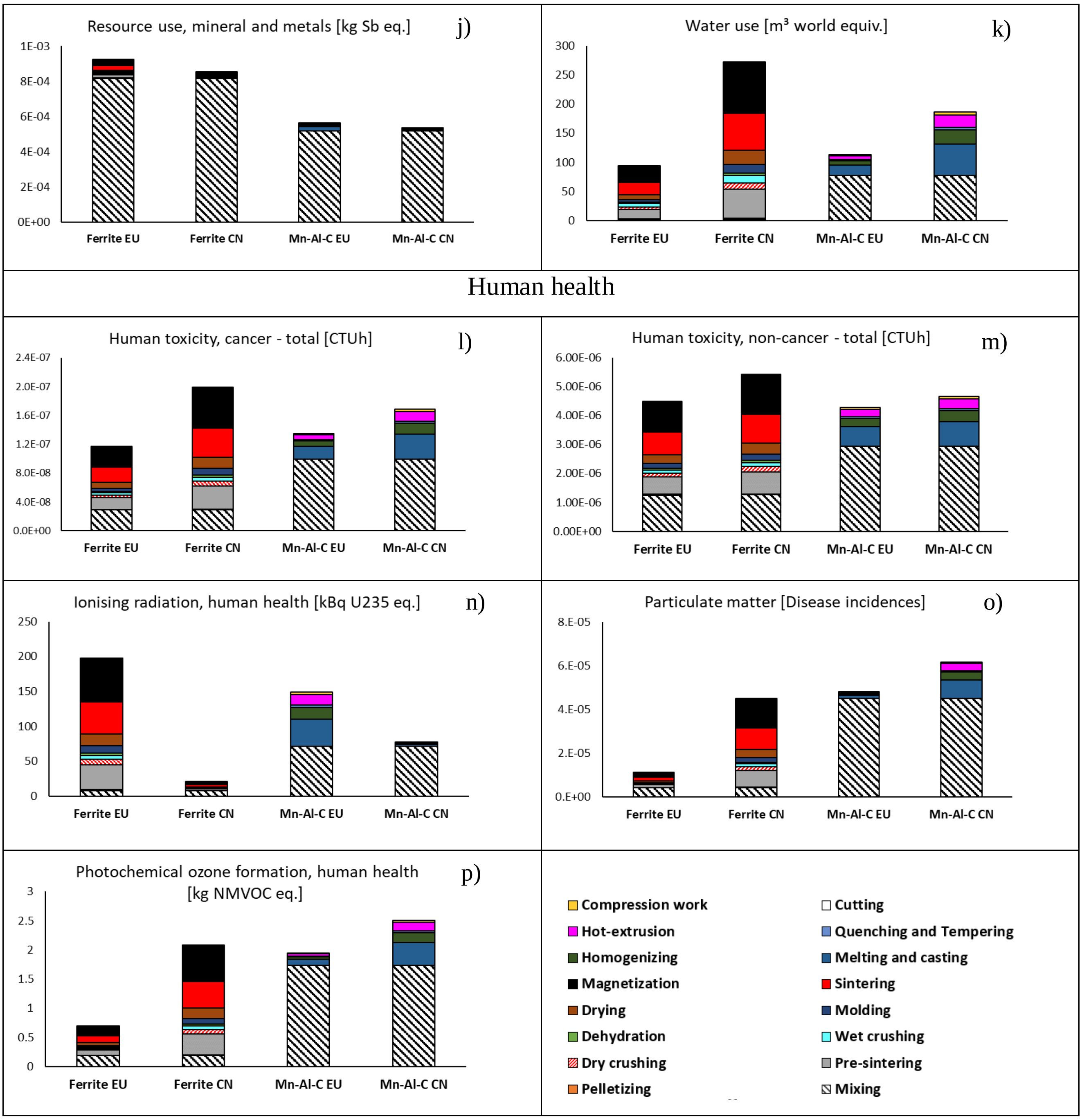

Figure S1 Assessment of environmental impacts in the categories of a) acidification, b) climate change, c) ecotoxicity, freshwater, d) eutrophication, freshwater, e) eutrophication marine, f) eutrophication terrestrial, g) ozone depletion, h) land use, i) resource use, fossils, j) resource use, mineral and metals, k) water use, l) human toxicity, cancer, m) human toxicity, non-cancer, n) ionizing radiation, human health, o) particulate matter, p) photochemical ozone formation, comparison between production processes of ferrite and Mn-Al-C magnets. Energy is supplied by Chinese grid mix (functional unit: 100 kg of magnets).

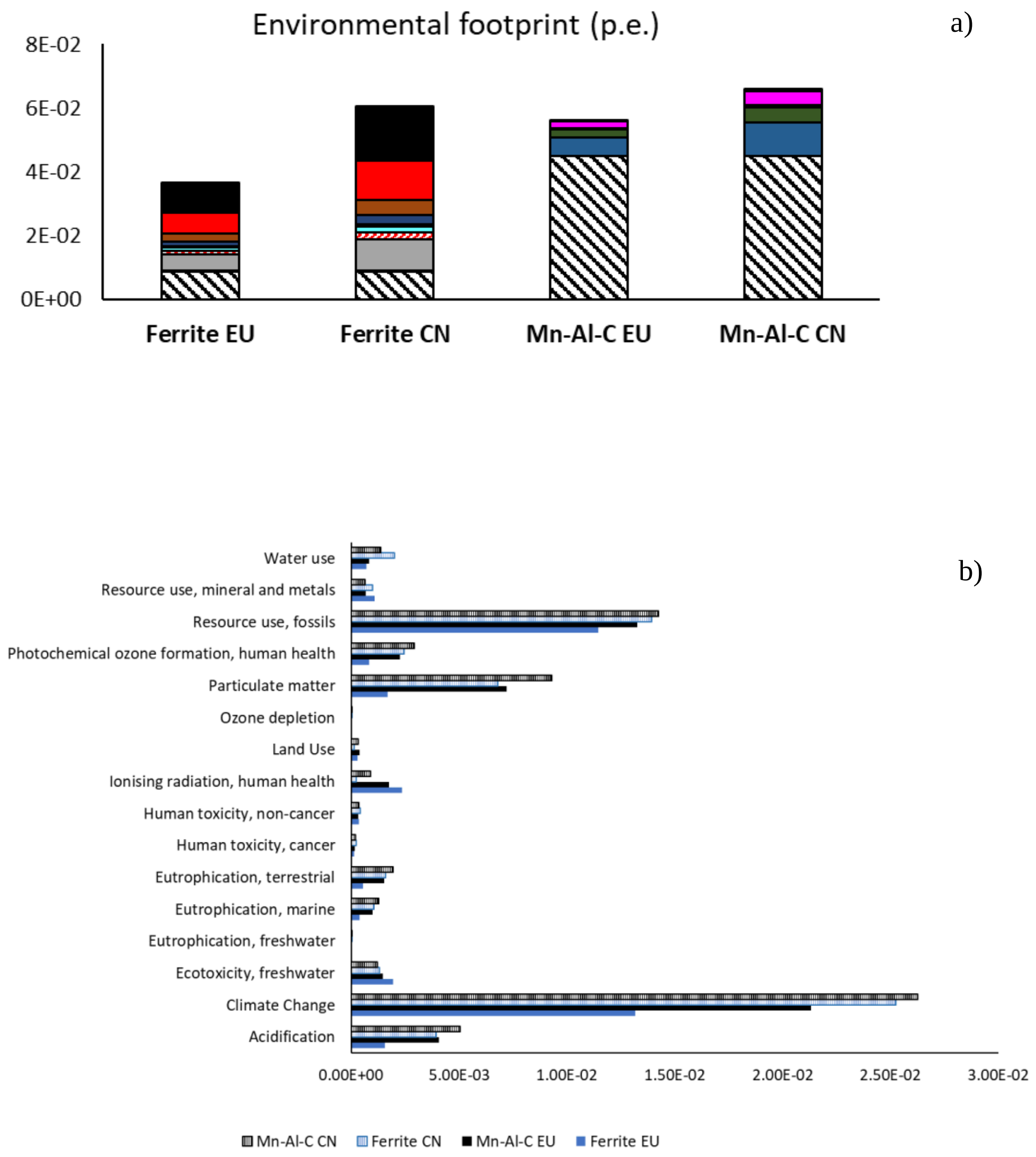

Figure S2: Assessment of environmental footprint: a) comparison between production processes of ferrite and Mn-Al-C magnets b) contribution of impact categories. Energy is supplied by Chinese grid mix (functional unit: 100 kg of magnets)

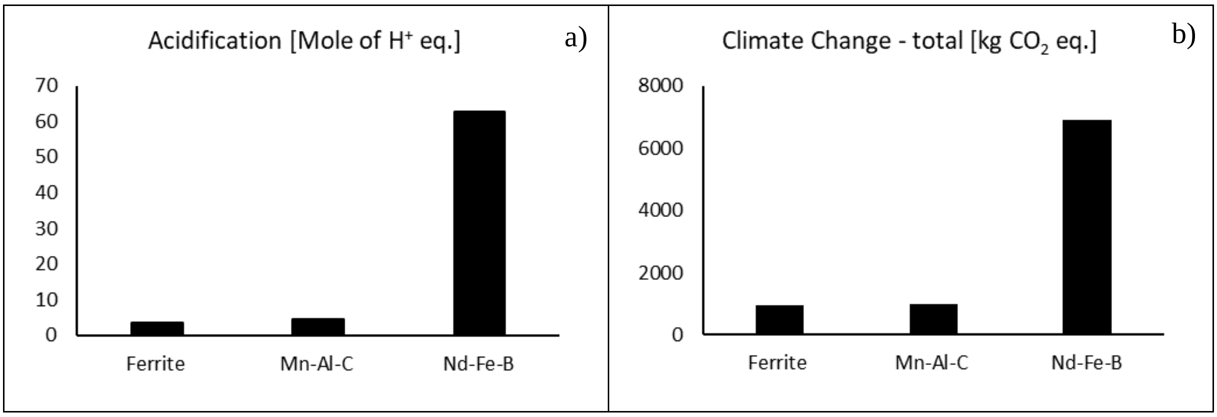

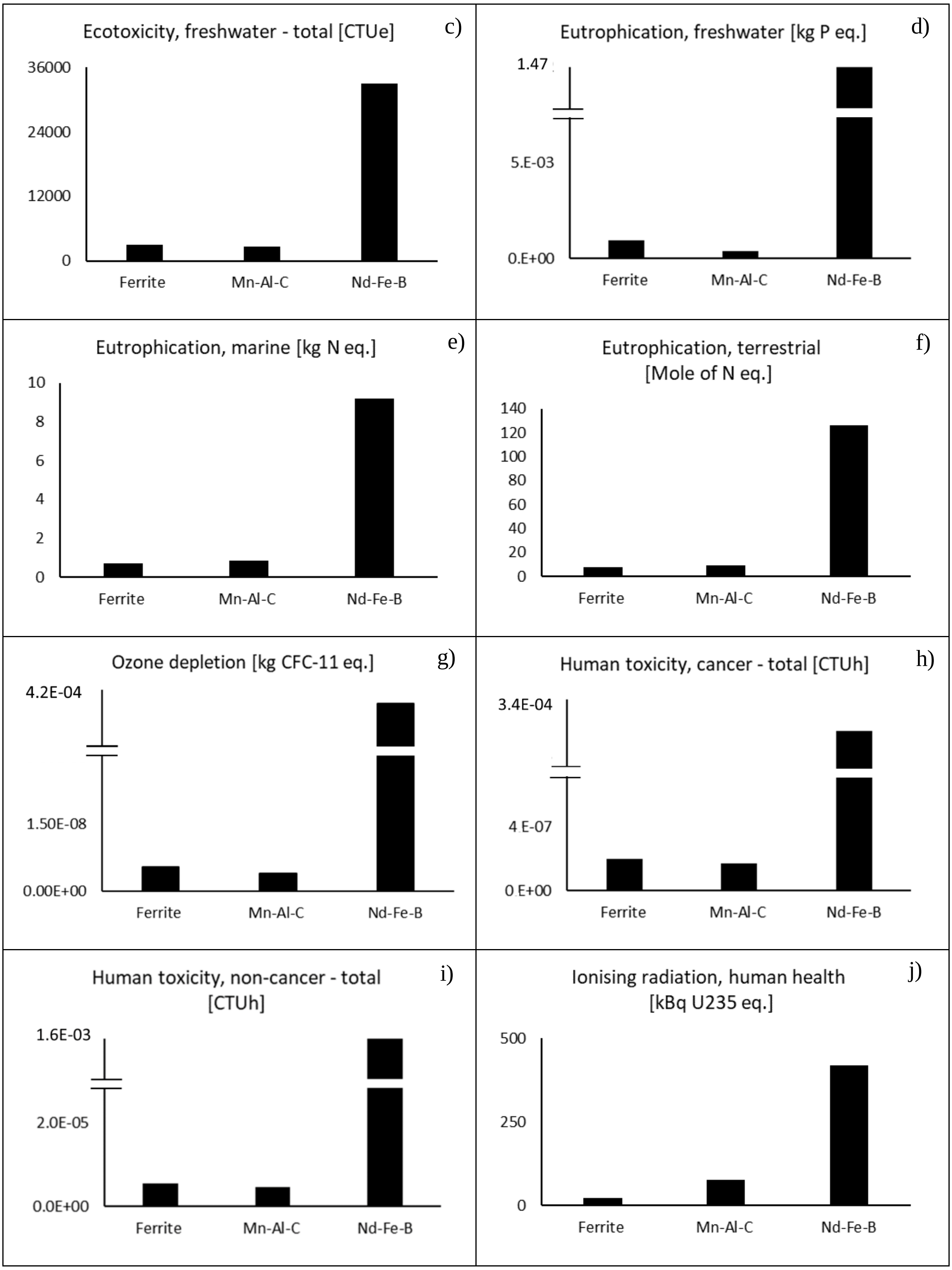

Ecotoxicity, freshwater - total [CTUe]
c)
36000
24000
12000
0
Ferrite
Mn-Al-C
Nd-Fe-B
Eutrophication, freshwater [kg P eq.]
d)
1.47
5.E-03
0.E+00
Ferrite
Mn-Al-C
Nd-Fe-B
Eutrophication, marine [kg N eq.]
e)
10
8
6
4
2
0
Ferrite
Mn-Al-C
Nd-Fe-B
Eutrophication, terrestrial [Mole of N eq.]
f)
140
120
100
80
60
40
20
0
Ferrite
Mn-Al-C
Nd-Fe-B
Ozone depletion [kg CFC-11 eq.]
g)
4.2E-04
1.50E-08
0.00E+00
Ferrite
Mn-Al-C
Nd-Fe-B
Human toxicity, cancer - total [CTUh]
h)
3.4E-04
4 E-07
0 E+00
Ferrite
Mn-Al-C
Nd-Fe-B
Human toxicity, non-cancer - total [CTUh]
i)
1.6E-03
2.0E-05
0.0E+00
Ferrite
Mn-Al-C
Nd-Fe-B
Ionising radiation, human health [kBq U235 eq.]
j)
500
250
0
Ferrite
Mn-Al-C
Nd-Fe-B

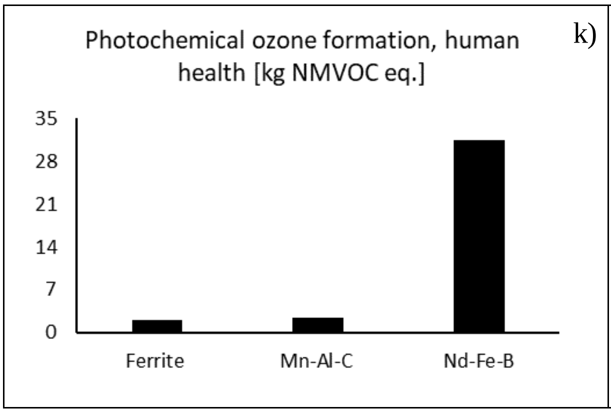

Figure S3: Environmental impact assessment of manufacturing processes in the categories of a) acidification, b) climate change, c) ecotoxicity, freshwater, d) eutrophication, freshwater, e) eutrophication marine, f) eutrophication terrestrial, g) ozone depletion, h) human toxicity, cancer, i) human toxicity, non-cancer, j) ionizing radiation, human health, k) photochemical ozone formation, comparison among sintered ferrite, Mn-Al-C and Nd-Fe-B magnets. Energy is supplied by Chinese grid mix.

References

(1) Amixon mixing technology. *Conical mixer Type AM*. https://www.amixon.com/en/products/conical-mixer (accessed 2023-07-14).

(2) Telang. *Activated alumina ceramic ball pelletizer machine*. https://www.telangmachine.com/ (accessed 2023-07-14).

(3) Agico Cement. *Electric heating rotary kiln*. https://www.rotarykilnfactory.com/electric-heating-rotary-kiln/ (accessed 2023-07-14).

(4) Tencan. *380V Rolling Ball Mill Ultra Fine Powder Milling with Alumina Ceramic Liner Cylinder*. https://www.laboratoryballmill.com/sale-10839488-380v-rolling-ball-mill-ultra-fine-powder-milling-with-alumina-ceramic-liner-cylinder.html (accessed 2023-07-14).

(5) Alibaba. *Solid-Liquid Semi-Automatic separator Machine Centrifuge*. https://www.alibaba.com/product-detail/Solid-Liquid-Semi-Automatic-separator-Machine_62185577074.html (accessed 2023-07-14).

(6) Dexing Magnet. *DXBT-100 Magnetic Field Injection Machine*. https://www.magnetomachinery.com/magnet-field-injection-machine-2.htm (accessed 2023-07-14).

(7) Labocon. *Oven*. https://www.labocon.com/catalog/oven.pdf (accessed 2023-07-14).

(8) Nordelöf, A.; Grunditz, E.; Lundmark, S.; Tillman, A. M.; Alatalo, M.; Thiringer, T. Life Cycle Assessment of Permanent Magnet Electric Traction Motors. *Transp. Res. Part D Transp. Environ.* **2019**, *67*, 263–274, DOI 10.1016/j.trd.2018.11.004.

(9) Sprecher, B.; Xiao, Y.; Walton, A.; Speight, J.; Harris, R.; Kleijn, R.; Visser, G.; Kramer, G. J. Life Cycle Inventory of the Production of Rare Earths and the Subsequent Production of NdFeB Rare Earth Permanent Magnets. *Environ. Sci. Technol.* **2014**, *48* (7), 3951–3958, DOI 10.1021/es404596q.

(10) Induction, 5M. *Induction melting/heating/holding*. http://5mtr.com/wp-content/uploads/2020/06/5M-Catalogue.pdf (accessed 2023-07-14).

(11) Nabartherm. *Thermal process technology*. https://nabertherm.com/sites/default/files/2021-

01/thermalprocesstechnology_english.pdf (accessed 2023-07-14).

(12) China extrusion press. *Extrusion press ABE-630*. http://www.china-extrusion-press.com/extrusion-press/Extrusion-Press-ABE-630.htm (accessed 2023-07-14).

(13) Alibaba. *Automatic mechanical powder compacting press machine*. https://cnhailichina.en.alibaba.com/product/62145089149-215450835/3_ton_press_machine_HPP_30F_automatic_Mechanical_powder_compacting_press_machine.html (accessed 2023-07-14).
